# Accessing Care: Experiences of Minoritised Ethnic Communities

**DOI:** 10.55975/TWNH7196

**Published:** 2025-11-01

**Authors:** H Richards, L Parsons, E Heeley, J Hill, C Harris

**Affiliations:** Liverpool Women’s NHS Foundation Trus; Picton & Kensington Children’s Centre; Mersey Care NHS Foundation Trust; University of Lancashire

## Introduction

Women from ethnic minority groups are at a higher risk of adverse outcomes in the antenatal period and experience higher maternal mortality rates compared with white women.^1^ In the UK, the rate of stillbirths is more than twice as high among babies of Black ethnicity and approximately one and a half times higher among babies of Asian ethnicity compared to white ethnicity.^2^ Good antenatal care can help prevent adverse outcomes and is important for health promotion, screening for and diagnosis of potential problems, and for the prevention and timely treatment of pregnancy related diseases.^3^ The National Institute of Health and Care Excellence guidance recommends that childbearing women and people have their first antenatal appointment by 10 weeks of gestation.^4^ However, women from ethnic minority groups are more likely to present late for antenatal care (after 12 weeks of gestation)^5^ and are less likely to have adequate utilization of antenatal care.^6^ Sharma et al., 2023 conducted a systematic review to examine the experiences of ethnic minority women accessing antenatal care in high-income European countries, aiming to develop a conceptual framework for understanding access to care.^7^

## Aim of commentary

This commentary aims to critically appraise the methods used within the review Sharma et al., 2023^7^ and expand upon the findings in the context of clinical practice.

## Methods

This protocol registered qualitative review involved a literature search of seven databases (PubMed, PsycINFO, Scopus, CINAHL, Medline, Global Health, and the Cochrane Library) from date of inception to February 2021. Only qualitative or mixed methods studies, published between 2010-2021 in English, which explored access to antenatal care for ethnic minority women in high income European countries were included. Title and abstract, and then full text screening was undertaken independently by two authors with arbitration by a third. Data extraction and quality assessment, (Critical Appraisal Skills Programme checklist) for qualitative studies, were conducted by two reviewers; however, it is unclear whether these tasks were performed independently. Data analysis was conducted using a “best fit” framework synthesis in which a theoretical framework was identified a priori, which the extracted data was coded against.

## Results

Following the screening process 30 studies were found to meet the inclusion criteria and were included in the synthesis. The included studies were conducted in 11 high-income European countries. Study participants were from ethnic minority and/or migrant groups. Twelve of the studies identified where participants had arrived recently in their host countries within the past five years.

In the quality assessment, almost all the included studies were judged as using appropriate methods to address the studies’ aims, and over half (n=16) justified the choice of research design. The main methodological issue identified was a lack of description of the relationship between the researchers and the participants in the majority of included studies (n=28). Furthermore, many of the studies did not provide adequate detail around recruitment methods (n=19), data collection methods (n=17), or data analysis processes (n=24).

A synthesis of the findings was conducted using a “best fit” framework based on Levesque’s model of access to healthcare, with themes broadly grouped into two main themes: Provision of Antenatal Care (“supply side”) and Women’s Uptake of Antenatal Care (“demand side”).

The theme of provision of antenatal care was concerned with factors affecting access resulting from the way antenatal care is provided and included the sub-themes of promotion of antenatal care importance; making contact and getting to antenatal care; costs of antenatal care; interactions with antenatal care providers; and models of antenatal care provision. Within this theme women reported difficulties in understanding and navigating the health care system, the mechanisms and logistics of making antenatal appointments, and the cost and logistics of travelling to appointments. In some studies women reported experiencing frequent stigmatising and discriminatory interactions with care providers which could negatively impact on future uptake and access to care. Models of antenatal provision were seen to intersect with all aspects of access to care, with continuity of carer being identified as particularly valued by women or desired where it wasn’t available.

The theme of women’s uptake of antenatal care was concerned with women’s abilities to, and difficulties in accessing antenatal care. This included the sub-themes of delaying initiation of antenatal care; seeking antenatal care; help from others in accessing antenatal care; and engaging with antenatal care. Three further sub-themes were seen to cut across and impact on all aspects and levels of access to antenatal care: previous experiences of interacting with maternity services; ability to communicate; and immigration status. Within this theme employment flexibility was cited as a barrier to attending antenatal appointments with women reporting being afraid to ask for time off work particularly when working illegally or on short term contracts. Previous poor care (either maternity or general healthcare) was cited as having a negative effect on participants, resulting in a loss of trust and disengagement with antenatal services. Language and communication difficulties were reported to be significant barriers to accessing care, with problems cited including a lack of suitable interpreters and a lack of written information in women’s spoken languages. Uncertain immigration status was also identified as an important barrier with women expressing a fear of being reported to immigration authorities, or having children removed from them, when accessing antenatal care.

## Commentary

Using the Joanna Briggs Institute critical appraisal tool for systematic reviews^8^, 6 out of the 10 criteria that were applicable to this review were judged to be satisfactory. It was therefore deemed that the review might provide an accurate summary of the findings of the included studies. However, there were concerns that the searches were limited to papers published between 2010 and 2021, and papers in languages other than English were excluded, without justification for these restrictions. Since the review sought to synthesise ethnic minority women’s experiences of accessing antenatal care in European countries this may have excluded useful papers published in other languages. It is notable that seven of the included studies were identified through citation searching or reviewer recommendation rather than through the main database searches, which could indicate that the search strategy was not sufficiently sensitive enough to identify all relevant literature. It is also notable that 3719 of the 4025 records retrieved in the searches were removed as duplicates, and only 333 records were screened. It is therefore difficult to assess how comprehensive the findings of the review are.

There was also a lack of clarity around how the quality assessment and data extraction processes were conducted. Although the review states that two reviewers conducted the critical appraisal and data extraction of included papers it is unclear whether these tasks were done independently or not. Independent data extraction and critical appraisal by two reviewers is recommended in order reduce errors.^9,10^ Finally, there were concerns around the extent to which recommendations for policy and practice made by the authors were supported by the review findings which were felt to be highly subjective.

Evidence from this review highlights the importance of information and communication in enabling access to services for ethnic minority women. NICE guidance states that those responsible for the organisation of antenatal services should provide information in a variety of formats, settings, and languages.^11^ It is essential that information about how to find and use antenatal services is advertised in a variety of settings in the community such as pharmacies, community centres, faith centres, healthcare centres, hostels, and other accommodation.^11^ Healthcare organisations must ensure that professional interpreters are always offered rather than relying on family and friends, and children should never be used as interpreters.^12^ It is advisable that healthcare professionals check that patients are able to read, and sufficiently understand health information, in their preferred language before offering written materials.^13^ Audio-recorded spoken information could be an alternative to written information and images and diagrams may be useful where patients have low health literacy.^13^ Text messages, phone calls or translated letters may be a more effective way to contact patients than letters written in English.^12^

Continuity of carer was identified in the review as particularly valued or desired. Where possible it is preferable that ethnic minority women are provided with midwife-led continuity of care to improve maternity outcomes.^14^ Specialist antenatal services should be developed for vulnerable ethnic minority groups co-designed with the local community, third sector organisations and lay groups to ensure suitability and accessibility.^15^

The review findings suggest that ethnic minority women frequently experience stigmatising and discriminatory interactions with care providers which could negatively impact future uptake and access to care. This issue was similarly identified by the Birthrights inquiry^16^ into racial injustice and human rights in UK maternity care which found widespread reports of women and birthing people experiencing racism, disrespect, stereotyping and dehumanisation within the healthcare system.^17^ A study of midwives’ experiences of caring for ethnic minority women also reported that midwives expressed a lack of, and a need for, better training in cultural awareness.^18^ To address these issues healthcare providers must provide targeted cultural sensitivity training to staff, to increase awareness and to improve understanding of different cultural practices, preferences, and beliefs relating to pregnancy, childbirth, and postpartum periods.^18^ Unconscious bias and anti-racism training should be robust and mandatory to educate professionals on recognising and mitigating implicit biases that can affect care delivery, and to help foster an inclusive and respectful care environment.^18^

The review identifies barriers and facilitators to accessing care that ethnic minority women experience. Further research is needed to explore interventions and service developments to increase uptake and improve childbearing women and people’s experiences of antenatal care. Research is needed to establish whether guidance and recommendations around antenatal care for ethnic minority populations are being implemented into practice and to assess whether this is effective in improving access to care.

## Key Points

- Ethnic minority women report difficulties in understanding and navigating the healthcare system and experience stigmatising and discriminatory interactions with care providers which can affect uptake of care.
- Information and communication are important factors in enabling access to services for ethnic minority women.
- Where possible ethnic minority women should be provided with midwife-led continuity of care to improve maternity outcomes
